# Supplementary material for: Learning curve and surgical outcome of robotic assisted colorectal surgery with ERAS program
Source: Sci Rep. 2022 Nov 29;12:20566. doi: 10.1038/s41598-022-24665-w (PMC9709162; doi:10.1038/s41598-022-24665-w)
Supplement: Supplementary file 2 — Supplementary Information 2. [file 41598_2022_24665_MOESM2_ESM.zip › RAL Raw data-Ñ[▒K/075/HS_ñw╝╨░O▒Kñσ.pdf]

正式摘要(電子簽章：000000215426557)

W95 / W95 ☐英文標題(注意:無英文姓名-請補病人基本檔!) ☐【讀取】憑證

臺中榮民總醫院

出院病歷摘要

轉入號： 轉出號：

[redacted]

診斷

- 入院：1. Colon Cancer, c T3 N2 M0, Stage III
  - s/p neo-adjuvant CCRT
- 出院：1. Colon Cancer, c T3 N2 M0, Stage III
  - s/p neo-adjuvant CCRT
  - s/p Robot Assisted Low Anterior Resection+loop ileostomy 2021/04/22

主訴

- difficult defecation with bloody stool for one month

病史

- This 63 year-old male denied DM or HTN
  He felt anal swelling and difficulty in defecation for 2 month, and sometimes bloody stool. He went to Dr.CHEN OPD for help. On 2020/12/30, non-sedative colonoscopy up to 8cm FAV, rectal tumor with partial obstruction noted. Pathology showed highly suspect for adenocarcinoma. Neo-adjuvant CCRT was arranged. After CCRT, this time, he was admitted for surgical intervention.

體檢發現

-
  - General appearance :
    - easy looking , conscious clear , without cardiopulmonary distress
    - Vital signs自動帶入date 體溫(BT) 脈搏(P) 呼吸(R) 血壓(BP) 身高(BH) 體重(BW) BMI
      20210421 35.9℃ 81次/分 19次/分 140/90mmHg 153cm 49.5kg 21.1
    - Vital signs: B.P. - mmHg , P.R. 5 /min , R.R. 5 /min
  - HEENT :
    - Eyes: no anemic conjunctiva, no icteric sclera, normal EOM
    - ENT: no otorrhea, no rhinorrhea, no erythematous mucosa, no injected tonsil, no deformity
  - Neck: supple, freely movable, trachea midline,no deformity, no engorged jugular vein , no palpable thyroid or lymph node enlargement
  - Chest:
    - Inspection: Grossly normal, symmetric and fully expansion, no accessory muscle used, no operation scar, no spider angiomata, no gynecomastia
    - Palpation: no palpable mass or nodule, no subcutaneous emphysema, no tenderness
    - Ausculation: clear breathing sound
  - Heart:
    - Percussion: normal heart size
    - Ausculation: regular heart beat, normal S1, S2, no gallop, no murmur
  - Abdomen:
    - Inspection: in
      - flat contour, symetrically, no scar, no stria, no dilated veins, no rashes
    - Ausculation: normoactive bowel sound, no abdominal bruits
    - palpation: soft, no muscle guarding or rigidity, no local tenderness, no rebounding pain, no palpable mass, no palpable pulsation
    - Percussion: no shifting dulllness, no hepatomegaly, no splenomagaly
  -

Peripheral pulsation :	CA	BA	RA	FA	PA	PTA	DPA
Right side :	++	++	++	++	++	++	++/+++
Left side :	++	++	++	++	++	++	++/+++

  - Genito-anus: no tenderness or palpable mass lesion or rectal shelf, normal external genitalia, normal anal tone, brown stool

CRS CONDITION

-
  - Lung

手術日期及方法

- 手術日期及方法20210422 Robot Assisted Low Anterior Resection + loop ileostomy
- 手術結果及發現：
  - 1. ulcerative tumor, about 6 cm in size 6 cm FAV.

住院治療經過

- After admission, there was no contraindication for operation and Robot Assisted Low Anterior Resection +loop ileostomy was performed smoothly on 2021/04/22. He tolerated the procedures and no post-op complication was noted. Oral intake was smooth since 04/23. Under the stable condition, he was discharged and would be followed-up at OPD.

合併症

- Nil

一般檢查

- 累積報告 :

DATE	TIME	ALB	TP	BIL.T	BIL.D	ALKP	AST	ALT	LDH	CHOL	TG	HDL-C	LDL	UA	NH3	AMY
1100421	1723									188	62	50	123	5.3		
1100422	1436			0.55		86	19	22								

DATE	TIME	WBC	RBC	HGB	HCT	MCV	PLT	BLST	NEUT	BAND	SEG	LYM	MON	EOS	BAS
1100421	1716		4.34												
1100422	1436	9880	4.18	12.8	37.1	88.8	217		94.8			3.5	1.6	0.0	0.1

DATE	TIME	NA	K	CL	CA	BUN	CREAT	Mg	P	CRP	ESR	PCT	LIPASE	LACTATE
1100421	1723									0.123				
1100422	1435													10.9
1100422	1436	138	4.3			9	0.74							

DATE	TIME	GLUCOSE	PAB	TRF	ZN	CU	KETONE	CK	CKMB	PT-P	PT-C	APTT-P	APTT-C	TRO-T
1100421	1657	78												
1100421	1912									10.9	10.6	29.8		

- 放射線報告 :**檢驗項目 :MRI - PELVIS 報告日期 :1100407**

主　訴：
Result:Y
Finding:
Imaging Report Form for Colorectal Carcinoma (AJCC 8th)
1. Imaging Date: 20210405
Imaging Modality, Imaging by
MRI
Imaging Protocol: MRI study of pelvis was performed with pulse sequences of T2WI, DWI & T1WI (FS) after gadolinium injection
2. Tumor location / Size
Location:
Rectum
Size:
Measurable: 7.0 cm (largest diameter)
3. Tumor invasion
Tumor invades non-peritonealized pericolonic or perirectal tissues
4. Regional nodal metastasis
N1 | N1a One regional lymph node is positive.
5. Distant metastasis (In this study)
No or Equivocal
6. Other findings: 1) Presence of wall thickening of upper rectum with perirectal fat stranding, compatible with rectal cancer.
2) A 0.8cm nodule is noted at right perirectal region, regional lymph node is considered.
3) No other abnormal lymphadenopathy in pelvis or bilateral inguinal region.
Impression:
Compatible with rectal cancer

特殊檢查

病理報告

- Pending

其它

出院時情況

- 3.改門診治療

出院指示

藥名	劑量	劑量單位	用法	頻次	數量	數量單位	現況	天數	單位
(冂)Takepron OD tab 30mg	1	TAB	PO	QDAC	8	TAB	TH	8	天
Ultracet tab	1	TAB	PO	Q6H	32	TAB	TH	8	天

出院計劃

主治醫師 2062F 蔣鋒帆 / 住院醫師 2334J 陳勇先

正式摘要(**電子簽章：000000215426557)**

W95 / W95

doctorRank:B
